# Antiviral activity of stachyflin on influenza A viruses of different hemagglutinin subtypes

**DOI:** 10.1186/1743-422X-10-118

**Published:** 2013-04-16

**Authors:** Yurie Motohashi, Manabu Igarashi, Masatoshi Okamatsu, Takeshi Noshi, Yoshihiro Sakoda, Naoki Yamamoto, Kimihito Ito, Ryu Yoshida, Hiroshi Kida

**Affiliations:** 1 Department of Disease Control, Laboratory of Microbiology, Graduate School of Veterinary Medicine, Hokkaido University, Kita-18 Nishi-9, Sapporo 060-0818, Japan; 2 Division of Bioinformatics, Research Center for Zoonosis Control, Hokkaido University, Sapporo 001-0020, Japan; 3 Discovery Research Laboratories, Shionogi & Co., Ltd., Settsu, Osaka 566-0022, Japan

**Keywords:** Stachyflin, Anti-influenza drug, HA inhibitor, Docking model

## Abstract

**Background:**

The hemagglutinin (HA) of influenza viruses is a possible target for antiviral drugs because of its key roles in the initiation of infection. Although it was found that a natural compound, Stachyflin, inhibited the growth of H1 and H2 but not H3 influenza viruses in MDCK cells, inhibitory activity of the compound has not been assessed against H4-H16 influenza viruses and the precise mechanism of inhibition has not been clarified.

**Methods:**

Inhibitory activity of Stachyflin against H4-H16 influenza viruses, as well as H1-H3 viruses was examined in MDCK cells. To identify factors responsible for the susceptibility of the viruses to this compound, Stachyflin-resistant viruses were selected in MDCK cells and used for computer docking simulation.

**Results:**

It was found that in addition to antiviral activity of Stachyflin against influenza viruses of H1 and H2 subtypes, it inhibited replication of viruses of H5 and H6 subtypes, as well as A(H1N1)pdm09 virus in MDCK cells. Stachyflin also inhibited the virus growth in the lungs of mice infected with A/WSN/1933 (H1N1) and A/chicken/Ibaraki/1/2005 (H5N2). Substitution of amino acid residues was found on the HA2 subunit of Stachyflin-resistant viruses. Docking simulation indicated that D37, K51, T107, and K121 are responsible for construction of the cavity for the binding of the compound. In addition, 3-dimensional structure of the cavity of the HA of Stachyflin-susceptible virus strains was different from that of insusceptible virus strains.

**Conclusion:**

Antiviral activity of Stachyflin was found against A(H1N1)pdm09, H5, and H6 viruses, and identified a potential binding pocket for Stachyflin on the HA. The present results should provide us with useful information for the development of HA inhibitors with more effective and broader spectrum.

## Background

Influenza A virus is widely distributed in birds and mammals, including humans, and causes seasonal and pandemic influenza. For the prevention and therapy of influenza, anti-influenza drugs play an important role in addition to vaccination. Currently available anti-influenza virus drugs are M2 inhibitors (Amantadine and Rimantadine) and neuraminidase (NA) inhibitors (Oseltamivir, Zanamivir, Peramivir, and Laninamivir) [1,2]. The M2 transfers protons into the infecting virus in the endosome at low pH, and the M1 is dissociated from the genome-transcriptase complex [3]. M2 inhibitors block ion channel activity and inhibit the influx of protons, thereby exhibiting antiviral activity against influenza A viruses [1]. At the end of the virus life cycle, the NA catalyses the cleavage of terminal sialic acid from glycoconjugates on the host cell surface to release progeny virions [4]. Of these anti-influenza virus drugs, the NA inhibitors, which interfere with the release of the virus, are used clinically since they are broadly effective [5]; however, concern has been raised because of the isolation of NA inhibitor-resistant viruses from clinical samples [6]. Therefore, it is expected to develop drugs targeting other virus proteins than the NA and M2.

Hemagglutinin (HA) is a surface glycoprotein of influenza A virus, and is a possible target of antiviral drugs because of its key roles in the initiation of infection. Each monomer of the trimeric HA is composed of 2 subunits, HA1 and HA2. The HA1 has a receptor binding domain, and the HA2 mediates the fusion of the virus envelope with the cellular membrane [7]. Several studies have identified compounds which inhibit viral infection by blocking the binding of the HA to sialic acid receptor on the host cell surface (cyanovirin-N and trisphenol-sialyllactose) or fusion step (TBHQ, BMY-27709, CL-385319, and N-carboxamide) [8-12]; however, for many of these inhibitors, the antiviral spectrum is limited to the HA of certain subtypes, so that they have not been used clinically. To develop more effective HA inhibitors, further investigations of these HA inhibitors and the analysis of the attachment and fusion steps of influenza virus infection in the host cells are needed.

It was found that a sesquiterpene derivative, Stachyflin, inhibited replication of H1 and H2 influenza A viruses *in vitro*[13,14] and *in vivo*[15,16]. Although Stachyflin is postulated to inhibit the fusion step, its precise mechanism has not been clarified. In the present study, it is revealed that Stachyflin inhibit the growth of H1, H2, H5, and H6 influenza viruses by binding the site of the HA2 and preventing the HA from fusion of the virus envelope with cellular membrane.

## Results

### Antiviral activity of stachyflin in vitro and in vivo

The antiviral spectrum of Stachyflin was determined by measuring its inhibitory effect on the replication of 31 influenza virus strains of H1-H16 subtypes in Madin-Darby canine kidney (MDCK) cells. The antiviral effects were evaluated in various concentrations of Stachyflin up to 6.50 μM by virus-induced cytopathic effects (CPE). Stachyflin inhibited the replication of H1 including A(H1N1)pdm09 virus, H2, H5, and H6 subtype influenza virus strains, but not that of the other subtype strains. Susceptibility of the viruses to Stachyflin varied with the strains (Table 1). In all of the viruses tested, AWSN/1933 (H1N1) (WSN) showed the highest susceptibility to Stachyflin.

**Table 1:** Antiviral activity of Stachyflin on influenza A virus

**Subtype**	**Virus strain**	**EC**_**50 **_**(μM)**^**a**^
H1	A/WSN/1933 (H1N1)	0.05
	A/swine/Hokkaido/2/1981 (H1N1)	0.24
	A/Puerto Rico/8/1934 (H1N1)	0.49
	A/Narita/1/2009 (H1N1) pdm	1.95
H2	A/Singapore/1/1957 (H2N2)	0.16
H3	A/duck/Hokkaido/5/1977 (H3N2)	>6.50
H4	A/duck/Czech/1956 (H4N6)	>6.50
H5	A/Hong Kong/483/1997 (H5N1)	1.95
	A/whooper swan/Hokkaido/1/2008 (H5N1)	2.05
	A/duck/Hokkaido/Vac-1/2004 (H5N1)	0.86
	A/chicken/Ibaraki/1/2005 (H5N2)	0.17
	A/chicken/Taiwan/A703-1/2008 (H5N2)	>6.50
	A/whooper swan/Mongolia/3/2005 (H5N1)	4.70
	A/peregrine falcon/Hong Kong/810/2009 (H5N1)	>6.50
H6	A/turkey/Massachusetts/3740/1967 (H6N2)	0.53
	A/duck/Hokkaido/31/2010 (H6N2)	0.65
	A/gull/Tottori/105/1980 (H6N3)	0.65
	A/duck/Taiwan/4801/1990 (H6N5)	0.44
	A/duck/Vietnam/OIE-2574/2011 (H6N6)	>6.50
H7	A/turkey/Italy/4580/1999 (H7N1)	>6.50
	A/chicken/Netherland/2586/2003 (H7N7)	>6.50
H8	A/turkey/Ontario/6118/1968 (H8N4)	>6.50
H9	A/chicken/Yokohama/aq-55/2001 (H9N2)	>6.50
	A/Hong Kong/1073/1999 (H9N2)	>6.50
H10	A/chicken/Germany/N/1949 (H10N7)	>6.50
H11	A/duck/England/1/1956 (H11N6)	>6.50
H12	A/duck/Alberta/60/1976 (H12N5)	>6.50
H13	A/duck/Siberia/272PF/1998 (H13N6)	>6.50
H14	A/mallard/Astrakhan/263/1982 (H14N5)	>6.50
H15	A/duck/Australia/341/1983 (H15N8)	>6.50
H16	A/black-headed gull/Sweden/5PF/1999 (H16N3)	>6.50

^a^ The compound concentration producing 50% inhibition of virus replication, as estimated by microscopic scoring of the CPE. The data shown are the means of 3 experiments.

**Figure 1:**
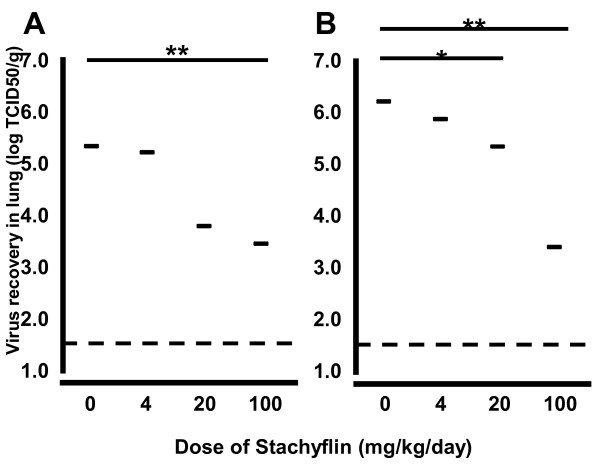
**Antiviral activity of Stachyflin in mice.** Four BALB/c mice were intranasally infected with 10 MID_50_ of WSN (**A**) or Ibaraki (**B**). After inoculation, the solution of Stachyflin in polyethylene glycol 400 was intraperitoneally administered to each group every 12 h for 72 h. At 72 h post-inoculation, mice were sacrificed and the lungs were collected for virus titration. Dashed lines indicate virus titer less than the detectable dose (10^1.5^ TCID_50_/g). *, *P*<0.05 compared to results for controls. **, *P*<0.01 compared to results for controls.

The antiviral activity of Stachyflin in mice challenged with A/Kumamoto/5/1967 (H2N2) was evaluated in previous study [15,16]. To confirm whether the antiviral activity *in vitro* can be applied to *in vivo*, the virus titers in the lungs of mice infected with WSN or A/chicken/Ibaraki/1/2005 (H5N2) (Ibaraki) 72 h post-inoculation were evaluated. WSN showed efficient replication and was lethal to mice, while infection with Ibaraki, a low pathogenic H5 avian influenza virus, was not lethal. Although mice treated with Stachyflin did not reduce the weight loss induced by virus challenge, only both groups of mice treated with Stachyflin at 100 mg/kg/day showed significantly lower mean virus titers in the lungs than each control group (Figure 1). In mice infected with WSN, the mean virus titer in the lungs of mice treated with Stachyflin at 100 mg/kg/day was 10^3.5^ 50% tissue culture infectious dose per gram (TCID_50_/g) whereas that of control mice was 10^6.2^ TCID_50_/g. In mice infected with Ibaraki, the mean virus titer in the lungs of mice treated with Stachyflin at 100 mg/kg/day was 10^3.4^ TCID_50_/g, whereas that of control mice was 10^5.4^ TCID_50_/g. Thus, Stachyflin showed inhibitory activity on virus growth *in vitro* and *in vivo*. In these experiments, CPE induced by Stachyflin was not observed even in the highest concentration, 6.50 μM in MDCK cells and weight changes of mice were not observed even in administration of 100 mg/kg/day for 3 days.

### Selection of stachyflin-resistant virus clones

To determine the amino acids which contribute to the susceptibility of the viruses to Stachyflin, Stachyflin-resistant virus clones were selected from WSN, A/Puerto Rico/8/1934 (H1N1) (PR8), Ibaraki, and A/duck/Taiwan/4801/1990 (H6N5) (Taiwan). Six Stachyflin-resistant virus clones were selected from WSN (WSN R1-R6) and 2 clones from PR8 (PR8 R1-R2) by single passage in MDCK cells in the presence of 0.52 or 1.30 μM of Stachyflin. The frequency of the Stachyflin-resistant virus clones was around 10^-3.0^-10^-4.0^. Meanwhile from Ibaraki and Taiwan, Stachyflin-resistant virus clones (Ibaraki R1 and Taiwan R1) were selected by 3 passages in the presence of 1.30 μM of Stachyflin. The clones were plaque-purified and propagated in MDCK cells in the presence of Stachyflin. The replication of these Stachyflin-resistant virus clones was not inhibited even with 6.50 μM Stachyflin (Table 2).

**Table 2:** The amino acid substitutions in the HA2 and character of Stachyflin-resistant (R) virus clones

**Virus**		**EC**_**50 **_**(μM)**	**Amino acid position in HA2**^**a**^							⊿**pH**^**c**^
			37	51	85	91	98	107	110	
WSN	Wild type	0.02	D	K	D	I	L	T	F	0.0
	R1	>6.50	N	-^b^	-	-	-	-	-	0.3
	R2	>6.50	-	R	-	-	-	-	-	−0.3
	R3	>6.50	-	-	H	-	-	-	-	0.0
	R4	>6.50	-	-	-	-	-	I	-	0.2
	R5	>6.50	-	-	-	-	V	-	-	−0.2
	R6	>6.50	-	-	-	F	-	-	-	0.0
PR8	Wild type	0.49	D	K	D	I	L	T	F	N.D.^d^
	R1	>6.50	-	-	-	-	S	-	-	N.D.
	R2	>6.50	-	-	-	-	-	-	S	N.D
Ibaraki	Wild type	0.17	D	K	D	I	L	T	F	N.D
	R1	>6.50	-	R	-	-	-	-	-	N.D.
Taiwan	Wild type	0.44	D	K	D	I	L	T	F	N.D.
	R1	>6.50	-	R	-	-	-	-	-	N.D.

^a^ H3 subtype numbering.

^b^ Dash (-) means the same amino acid as the wild type virus.

^c^ The pH at which 50% hemolysis of the wild type virus is 6.0. The values indicate the difference of the pH at which 50% hemolysis between the Stachyflin-resistant virus clones and wild type virus.

^d^ Not determined.

**Figure 2:**
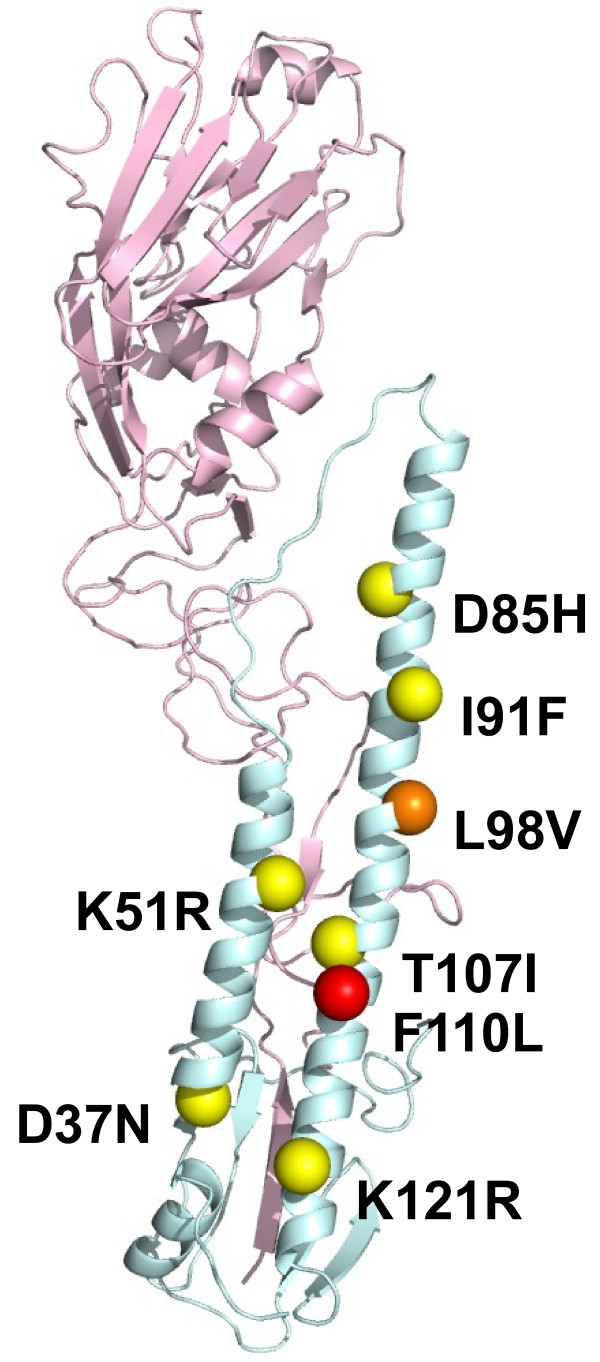
**Schematic representation of the positions of amino acid substitutions involved in Stachyflin resistance.** Three-dimensional image of the H1 HA molecule was created with data from X-ray crystallography of PR8 (PDB code: 1RU7) in the Protein Data Bank Japan and Discovery Studio Visualizer 1.6. Yellow spheres on the HA molecule indicate the positions of amino acid substitutions in Stachyflin-resistant virus clones of WSN selected in the presence of Stachyflin, and red sphere indicates that of PR8. Orange sphere indicates the position of amino acid substitution observed in both Stachyflin-resistant virus clones of WSN and that of PR8. The positions of amino acids correspond to the H3 HA numbering.

Nucleotide sequences of the HA genes of the wild type and Stachyflin-resistant virus clones were determined. All of the mutants had a single amino acid substitution in the HA2 (Table 2). The number of amino acid residue of HA2 starts from GLF motif and is common in these strains. Of these amino acid substitutions, K51R was common in the HAs of Stachyflin-resistant virus clones of WSN, Ibaraki, and Taiwan. These amino acid substitutions were mapped on the structure of the HA monomer (Figure 2). Although each of the amino acid substitutions was observed in the stem region of the HA2, it was impossible to form a binding site for Stachyflin by the distance. To confirm that each single mutation was responsible for Stachyflin resistance, rgWSN mutants, which have one amino acid substitution of the mutants, were generated by reverse genetics and site-directed mutagenesis and were characterized. The replication of rgWSN mutants was not inhibited by 6.50 μM Stachyflin, indicating that all the amino acid substitutions were responsible for Stachyflin resistance (Table 3).

**Table 3:** Character of rgWSN and rgStachyflin-resistant virus clones

**Virus**		**EC**_**50 **_**(μM)**	**Amino acid position in HA2**^**a**^			
			37	51	85	107
rgWSN	Wild type	0.02	D	K	D	T
	rgR1	>6.50	N	-^b^	-	-
	rgR2	>6.50	-	R	-	-
	rgR3	>6.50	-	-	H	-
	rgR4	>6.50	-	-	-	I

^a^ H3 subtype numbering.

^b^ Dash (-) means the same amino acid as the wild type virus.

### Optimal pH for hemolysis of stachyflin-resistant virus clones

Influenza virus mediates the hemolysis of chicken red blood cells (cRBC), which has been thought to represent the fusion of the virus envelope with cellular membrane [17]. Using a hemolysis assay, the effect of Stachyflin on the fusion of WSN wild type and Stachyflin-resistant virus clones was assessed. Stachyflin inhibited the hemolysis of cRBC induced by the wild type virus but not that by the mutants. In addition, optimal pH for fusion, at which 50% hemolysis occurred, shifted from 6.0 for the wild type virus to as follows: WSN R1: 6.3, R2: 5.7, R4: 6.2, and R5: 5.8 (Table 2).

### Relationship of amino acid substitution and the structure of potential binding pocket for stachyflin in the HA

**Figure 3:**
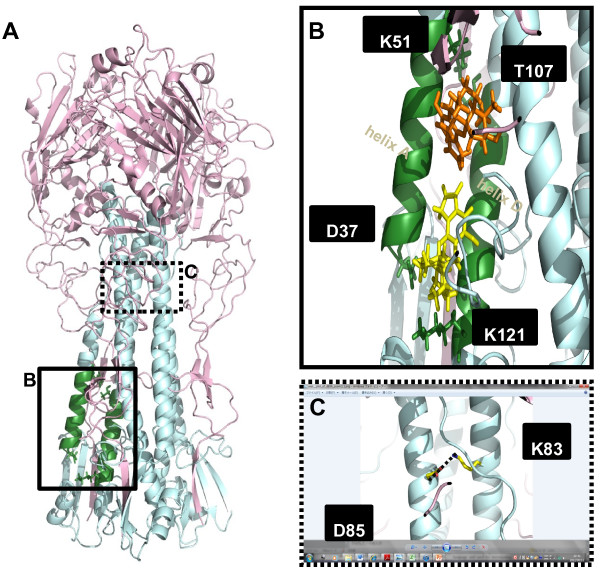
**The predicted docking model of Stachyflin with the H5 HA of Ibaraki.** Three-dimensional image of the HA trimer of Ibaraki was created based on the data from X-ray crystallography of A/Vietnam/1194/2004 (H5N1) (PDB code: 2IBX), and the sequence data of Ibaraki by homology modeling. (**A**) Residues colored in green indicate the region of the binding pocket for Stachyflin. The binding pocket is predicted to exist between helix A and helix D of the HA2 subunit and be surrounded by hydrogen bonds of D37-K121 and K51-T107, D37 to K51, and T107 to K121 residues in the HA2. (**B**) Binding position of Stachyflin in the binding pocket of the HA was predicted by docking simulation in Molegro Virtual Docker. The structure of Stachyflin is colored in yellow or orange and the residues constructing the binding pocket are in green. Two possible docking poses of Stachyflin with the HA were obtained, which are indicated as the positions of orange-colored Stachyflin (above) and yellow-colored Stachyflin (below) in the HA model. In the binding pocket, D37 may make a water-intermediate hydrogen bond with K121, and K51 may make a hydrogen bond with T107. (**C**) Dashed line indicates the salt bridge between D85 and K83 of another HA2 subunit. The distance between these residues was 2.55 Å.

To predict the possible docking model for Stachyflin in the HA trimer of WSN, PR8, Ibaraki, and Taiwan, computer docking simulations of Stachyflin with these HAs were performed. On the surface of these HA trimers, first, the binding pocket for Stachyflin was supposed to be located in a large cavity on the HA2 region, because most amino acid substitutions found on the HA of Stachyflin-resistant virus clones were in this cavity. In the cavity, we found a potential binding pocket for Stachyflin, which was formed by helix A and helix D of the HA2 subunit (Figure 3A, B). This binding pocket contained the residues, D37, K51, and T107, which were substituted in the HAs of Stachyflin-resistant virus clones (Figure 3B). In addition, a residue identified as a Stachyflin-resistant mutation previously [14], K121, was also contained in the region of the binding pocket (Figure 3B). It was also found that K51 and T107 made a hydrogen bond between helix A and helix D, which may stabilize the structure of the binding pocket.

Using computer docking modeling, it was investigated that how Stachyflin makes bonds with the amino acid around the binding pocket. In the present study, 2 possible docking models of Stachyflin and the HA were proposed (Figure 3B). In one model represented by orange-colored Stachyflin, Stachyflin bound to the site in the vicinity of T107 in the binding pocket, which is similar to that in a previous report [18] (Figure 3B). In the other model represented by yellow-colored Stachyflin, Stachyflin bound directly to D37 and K121 (Figure 3B). Both models were different from that in a previous study which postulated that Stachyflin forms a hydrogen bond with both K51 (helix A) and K121 (helix D) [14].

## Discussion

Anti-influenza virus drugs are important for the prevention and treatment of seasonal and pandemic influenza. The HA inhibitor is a candidate drug which inhibits virus attachment to or penetration into the host cells. Most fusion inhibitors hitherto reported had H1 and H2 or H3 subtype-specific antiviral activity and have not been examined their activities against H4-H16 viruses [10,11,19], except for CL-385319 [20]. Stachyflin was also reported as H1 and H2 subtype-specific fusion inhibitor and its 50% inhibitory concentration (IC_50_) was 0.2-0.6 μM [13]. The results of the present study revealed that Stachyflin had subtype-specific antiviral activities against not only H1 and H2 viruses, but also H5, including highly pathogenic avian influenza viruses, and H6 viruses, as well as A(H1N1)pdm09 virus in MDCK cells and its IC_50_ was 0.05-4.7 μM (Table 1). It is revealed that cytotoxicity of Stachyflin to the MDCK monolayers did not appear up to a concentration of 75 μM [13], however, for its insolubility [16], antiviral activity *in vitro* was assessed up to a concentration of 6.5 μM. In the present study, it was found that WSN strain was the most susceptible to Stachyflin, and then Ibaraki, an avian H5N2 virus isolated from chicken. Then, antiviral activities of the compound were evaluated against these viruses in a mouse model. It was previously revealed that the activity of Stachyflin was limited as about 40% of viruses were recovered from lungs of mice injected intraperitoneally with 2 mg/mouse/day (about 100 mg/kg/day) of Stachyflin compared to non-injected mice after the challenge of A/Kumamoto/5/1967 (H2N2) and 400 mg/kg/day of Stachyflin by intraperitoneal injection was not toxic to mice [15,16]. Stachyflin showed antiviral activity to reduce 10^2.0–3.0^ virus titer in lungs of mice against H1 and H5 viruses at a dose of 100 mg/kg/day. NA inhibitors, which are used clinically, showed efficient antiviral activity in mice at a dose of 20 mg/kg/day [21] whereas Stachyflin showed the same effect at a dose of 100 mg/kg/day, which is considered an overdose; therefore, in addition to the poor pharmacokinetic of Stachyflin [15,16] and limited spectrum, it may be difficult to apply Stachyflin in clinical use in the present form. However, Stachyflin may be clinically used in combination with some NA inhibitor such as Oseltamivir.

**Figure 4:**
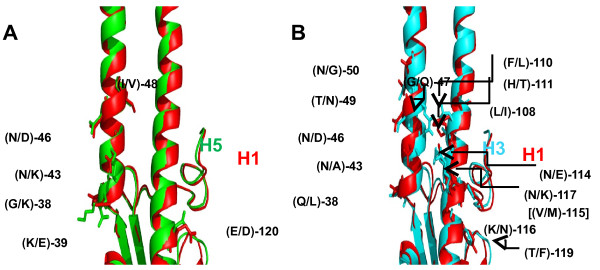
**Structural difference between the H1 and H5 or H3 HA in the binding pocket.** The amino acid residues indicated on the HAs differ between the H1 (PR8) and H5 (A/Vietnam/1194/2004 (H5N1)) or H3 (A/Aichi/2/1968 (H3N2)) (PDB code: 3HMG) HAs around the region of the binding pocket for Stachyflin. For example, (N/D)-46 indicates that residue 46 in the HA2 is asparagine in H1, but aspartic acid in H5 (**A**) or H3 (**B**). (**A**) The structure and side chains of the H1 HA are in red, and those of the H5 HA are in green. The 2 structures were overlapped and compared. (**B**) The structure and side chains of the H3 HA are in blue. [(V/M)-115] indicates that residue 115 in the HA2, which is valine in H1, but methionine in H3, is not visible.

Antiviral activity of Stachyflin was related with the structure of the HA. The structure of H1, H2, and H5 HAs, which are susceptible to Stachyflin, closely resemble each other [22] and these HAs including H6 were identified as group 1 (H1, H2, H5, H6, H8, H9, H11, H12, H13, and H16) by phylogenetic groupings of HA. The viruses used in this study have a similar sequence and structure of the binding pocket for the compound on the HA; for example, the structure of the binding pocket of the H1 HA is similar to that of the H5 HA compared to that of the H3 HA (Figure 4A, B). There are 6 different amino acids between the H1 and H5 HA around this region (Figure 4A). In particular, inside the binding pocket, only one amino acid at position 43 in the HA2 is different: WSN: asparagine, Ibaraki: lysine, which is assumed to cause the difference in the susceptibility to Stachyflin due to the difference in the size and charge of their side chains. For example, lysine has a larger side chain than asparagine and may make it more difficult for Stachyflin to enter into the binding pocket; therefore, the susceptibility to Stachyflin of Ibaraki was lower than that of WSN (Table 1). On the other hand, the HAs of the virus strains insusceptible to Stachyflin have different amino acid sequences in the binding pocket from that of the susceptible ones. For instance, 14 amino acids were different between the H1 and H3 HAs in the vicinity of the binding pocket, which cause a structural difference between these HAs (Figure 4B).

In the previous reports, 2 docking models of the HA and Stachyflin were suggested [14,18]. In one of the models, Stachyflin was postulated to make hydrogen bonds with K51 and K121 in the HA2 directly. The structure of the H3 HA, which is not susceptible to Stachyflin, was used to make the docking model; therefore, the model differed from our model, which uses the HA of susceptible viruses to Stachyflin. The other docking model was similar to one of those proposed in the present study, which is indicated as orange-colored Stachyflin (Figure 3B). However, since the software program for docking simulation was different from that used in the present study, several differences were found between these models. Indeed, the model which differed from both the previous docking models was also shown in the present study. In this study, we were unable to judge which of these models is more feasible. To further clarify these discrepancies, it is necessary to perform an X-ray crystallographic analysis of Stachyflin complex with the HAs to define the binding site for Stachyflin.

In most studies of HA inhibitors, mutations found in the HAs of inhibitor-resistant virus are explained as the cause to reduce their binding affinity with the compound and stabilization of the HA [10,19]. In the present study, it was indicated that D37, K51, D85, I91, L98, and T107 were involved in binding affinity with Stachyflin of the HA by the selection of Stachyflin-resistant virus clones. On the basis of the computer modeling in this study, K51 and T107 are postulated to make a hydrogen bond, which may stabilize the structure of the binding pocket for Stachyflin; therefore, mutations of these amino acids should lead to loss of this hydrogen bond, which may destabilize and distort the binding pocket and decrease the binding affinity of the HA to Stachyflin. Indeed, we selected Stachyflin-resistant virus clones which have the amino acid substitutions, K51R and T107I. In addition, we also identified amino acid substitutions, D37N and K121E, which are located close to each other on the HA. Interestingly, some of the crystal structure of the HA shows the possibility that D37 and K121 make a hydrogen bond via a water molecule. Then, similar to K51 and T107, the binding between D37 and K121 stabilizes the structure of the binding pocket and mutations of these amino acids lead to distortion of the binding pocket,decreasing the binding affinity of the HA to Stachyflin. Indeed, it was indicated by shift of fusion pH of the mutants that the amino acid substitutions, D37N, K51R, and T107I, may change the stability of the HA [22] (Table 2). The other possibility is that D37 and K121 are predicted to bind directly to Stachyflin based on the computer docking model (Figure 3B).

Although mutations of D85H, I91F and L98V were responsible for Stachyflin resistance, their locations on the HA were far from the region of the binding pocket for Stachyflin, leading us to investigate the effect of these mutations. Three-dimensional structure analysis showed that D85 and K83 of another HA2 subunit made a salt bridge, which stabilizes the structure of the HA strongly (Figure 3C) [22]. Amino acid substitution of D85H may abolish the interaction of the salt bridge and cause structural changes and destabilization of the HA, including the binding pocket. In addition, the substitution of aspartic acid to histidine causes structural and pH-dependent instability of the HA because of its electrostatic force [23]. Histidine collects protons around its side chain under low pH conditions, which might cause electrostatic repulsion between the 2 subunits [23]. Additionally, the Stachyflin-resistant virus clone with an amino acid substitution of D85H showed a weak hemolysis of cRBC, which may indicate its structural destabilization; therefore, it is assumed that the structural change of the HA occur by D85H, then Stachyflin is unable to bind to the HA of the D85H mutant.

The mutations of I91F and L98V are distant from the predicted binding pocket for Stachyflin whereas the residue of L98 is reported to be involved in the formation of the binding pocket for TBHQ, which is located upper that for Stachyflin, and have a hydrophobic interaction with TBHQ [19]; however, our docking simulation showed that Stachyflin was not likely to enter into the cavity for TBHQ (data not shown), suggesting that L98 may not interact directly with Stachyflin. Likewise, I91 may not interact with Stachyflin directly since there is no cavity around I91; therefore, amino acid substitutions of L98V and I91F are postulated to conformational change the structure of the HA, leading to a change in the structure or stability of the binding pocket for Stachyflin.

The frequencies of the Stachyflin-resistant virus clones selected from WSN and PR8 were 10^-3.0^-10^-4.0^. RNA viruses lack the ability to detect and repair mistakes during replication and, as a result, the mutation rate can be as high as 1 mutation per each 10^3^-10^5^ bases copied per replication cycle [24]. Based on these data, selection of these resistant variants was not ‘rapid’ but same as other drugs. Resistant variants from Ibaraki and Taiwan were selected in the presence of Stachyflin by 3 passage, indicating that the frequency of the virus clones were much lower than those of WSN and PR8. Structural analysis of the HA indicated that the lower stability of the HA by amino acid substitution for the Stachyflin-resistant virus lead to inefficient virus replication. These results indicate that the resistant virus clones exist in virus population and were isolated only under limited conditions.

## Conclusion

In the present study, we found antiviral activity of Stachyflin against A(H1N1)pdm09, H5, including highly pathogenic avian influenza viruses, and H6 viruses, and identified a potential binding pocket for Stachyflin, which differs from that previously proposed [12,14]. We hereby propose that molecular structures in the potential binding site for Stachyflin depend on the HA of different subtypes, affecting the susceptibility to this compound. Additionally, the present results suggest that further precise analysis of fusion inhibitors reveals their unidentified activities and more suitable docking poses with the HA, contributing to the further development of effective and broad-spectrum fusion inhibitors.

## Materials and methods

### Compound

Stachyflin obtained from *Stachybotrys* sp. has already been purified and characterized at Discovery Research Laboratories, Shionogi & Co., Ltd [25]. Stachyflin powder was dissolved in dimethyl sulfoxide (DMSO) (Nacalai Tesque, Kyoto, Japan) and was further diluted in each test medium.

### Cells and viruses

MDCK cells [26] were grown in Minimum Essential Medium (MEM) (Nissui Pharmaceutical, Tokyo, Japan) supplemented with 0.3 mg/ml L-glutamine, 5% fetal bovine serum (SAFC Biosciences, Street Lenexa, KS, U.S.A.), 100 U/ml penicillin G, 0.1 mg/ml streptomycin, and 8 μg/ml gentamicin. Human embryonic kidney (293T) cells were maintained in Dulbecco's Modified Eagle's Medium (DMEM) (Life Technologies Japan, Tokyo, Japan) supplemented with 0.3 mg/ml L-glutamine, 10% FBS (Cambrex, Grand Island, NY, U.S.A.), and antibiotics. Both cell lines were maintained at 37°C in a 5% CO_2_ atmosphere. The influenza virus strains used in the present study are listed in Table 1. All viruses were propagated in the allantoic cavities of 10-day-old embryonated chicken eggs at 35°C for 30–48 h. Before the infectious allantoic fluids were harvested, the eggs were chilled at 4°C overnight, and the harvested allantoic fluids were stored at −80°C.

### In vitro antiviral assay

Anti-influenza virus activity of Stachyflin was evaluated by its inhibition of virus-induced CPE in MDCK cells. The virus was inoculated onto confluent monolayers of MDCK cells at the titer of 100 TCID_50_/ml and virus was adsorbed to the cells at 4°C for 1 h. Unbound viruses were removed by washing the cells with PBS. MEM containing 1.0% DMSO and various concentrations of Stachyflin, from 0.004 to 6.50 μM, were added to the cells and incubated at 35°C. After 72 h, antiviral activity was evaluated by virus-induced CPE and expressed as 50% effective concentration of the compound (EC_50_).

### In vivo antiviral assay

Under anesthesia, 30 μl of WSN or Ibaraki containing 10 50% mouse infectious dose (MID_50_) was intranasally inoculated into 4-week-old female BALB/c mice (Japan SLC, Shizuoka, Japan). For the anesthesia, a mixture of tiletamine hydrochloride (20 mg/kg) (United States Pharmacopeia, Rockville, MD, U.S.A.), zolazepam hydrochloride (20 mg/kg) (United States Pharmacopeia), and xylazine (20 mg/kg) (Bayer HealthCare, Leverkusen, Germany) was injected intraperitoneally into mice. After the virus inoculation, 100 μl of the solutions containing various concentrations of Stachyflin in polyethylene glycol 400 (Nacalai Tesque) were intraperitoneally administered to each group every 12 h for 72 h. Control mice were injected with only polyethylene glycol 400 after the challenge. At 72 h post-inoculation, mice were euthanized and the lungs were collected for virus recovery. The supernatants of 10% lung homogenates were inoculated onto confluent monolayers of MDCK cells and the virus titers were calculated using the method of Reed and Muench and expressed as TCID_50_/g of tissue samples [27]. All animal experiments were carried out in self-contained isolator units (Tokiwa Kagaku, Tokyo, Japan) at the BSL-2 or BSL-3 facility of the Graduate School of Veterinary Medicine, Hokkaido University, Japan. The institutional animal care and use committee of the Graduate School of Veterinary Medicine authorized this animal experiment (approval numbers: 10–1052) and all experiments were performed according to the guidelines of this committee.

### Selection and characterization of stachyflin-resistant virus clones

WSN, Ibaraki, PR8, and Taiwan were diluted 10-fold series and inoculated on MDCK cells in the presence of various concentrations (0.26, 0.52, 1.30, and 6.50 μM) of Stachyflin. After 72 hours incubation at 35°C, the supernatant of the highest dilution series in the wells in which CPE was observed was collected. EC_50_ of the viruses were determined as described above and their susceptibilities to the compound were evaluated compared with the parent virus. If the recovered virus did not show increase of EC_50_, the virus was passaged by same method. For cloning of the viruses, each passaged virus was inoculated on MDCK cells and the cells were then overlaid with MEM containing 1% bacto-agar (Becton, Dickinson, and Company, Franklin Lakes, NJ, U.S.A.) in the presence of 6.50 μM Stachyflin. After 48 h of incubation at 35°C, the cells were stained with 0.014% neutral red (Kanto Chemical, Tokyo, Japan) and the plaques were collected. Individual clones were incubated on MDCK cells in the presence of 6.50 μM Stachyflin at 35°C. After 72 h incubation, each supernatant was collected and stored at −80°C.

### Sequence analysis of virus genes

Viral RNA was extracted from the allantoic fluid of embryonated chicken eggs or the supernatant of MDCK cells by TRIzol LS Reagent (Invitrogen, Carlsbad, CA, U.S.A.) and reverse-transcribed with the Uni12 primer [28] and M-MLV Reverse Transcriptase (Invitrogen). The full-length cDNA of the 8 gene segments was amplified by polymerase chain reaction (PCR) with gene-specific primer sets reported previously [29] or designed in the present study. The sequences of primers designed in the present study are as follows: PB2-826F: GTTAGGAGAGCAACAGTATCAG, PB2-922R: CAGCTTGCTCTTCTGTTGG, PB1-1240F: GGAATGATGATGGGCATGTT, PB1-1472R: CATCAGACGATTGGAGACCG, PA-723F: CATTGAGGGCAAGCTTTCTC, PA-1110R: CATGTTCTCACCTAATGCCC. Direct sequencing of all 8 gene segments was performed using an auto sequencer, 3500 Genetic Analyzer (Applied Biosystems, Foster City, CA, U.S.A.). To identify amino acid substitutions which should contribute to the susceptibility of viruses, the nucleotide sequences of Stachyflin-resistant virus clones were proofread, and the deduced amino acid sequences were compared with the wild-type virus using GENETYX-WIN version 10 (Genetyx, Tokyo, Japan).

### Reverse genetics

WSN and their mutants were generated by reverse genetics (rg) according to the procedure reports [30,31], which were named rgWSN, rgR1, rgR2, rgR3, and rgR4, respectively (Table 3). Briefly, viral RNA was extracted and amplified by RT-PCR. The PCR product of each gene segment was cloned into pHW2000 plasmid [31]. Eight genome sets of plasmid were transfected to MDCK and 293T cells and incubated at 37°C for 30 h and then 35°C. After 48 h, rgWSN was collected. All of the collected viruses were propagated in MDCK cells at 35°C and collected after 48 h.

### Site-directed mutagenesis

Stachyflin-resistant virus clones with the amino acid substitutions were generated by site-directed mutagenesis as described previously [31]. Briefly, the residue of amino acid substitutions in the HA2 were introduced into the HA genes of WSN using a Quik-Change II site-directed mutagenesis kit (Agilent, Santa Clara, CA, U.S.A.) according to the manufacturer’s instructions. The mutant viruses, rgR1, rgR2, rgR3, and rgR4, were rescued by reverse genetics as described above, and the entire genomes of the 8 gene segments were sequenced to confirm the existence of the introduced mutations and the absence of undesired mutations.

### Hemolysis assay

Hemolysis assay was performed as described previously [32]. Briefly, WSN and Stachyflin-resistant virus clones were centrifuged at 25,000 rpm for 1.5 h and the pellets were resuspended in PBS (pH 7.2). Virus concentrates were added to 1 ml of 1% cRBC in saline buffered with 0.1 M citric acid-sodium citrate at a final concentration of 200 HA unit and incubated on ice for 1 h. After the incubation at 37°C for 1 h with mixing every 10 min, the cells were sedimented by centrifugation and the supernatants were measured for hemoglobin at 540 nm.

### Protein and ligand structures

Three dimensional models of the H1 HA (WSN) and H5 HA (Ibaraki) molecules were constructed based on the HA crystal structures of PR8 and A/Vietnam/1194/2004 (H5N1), respectively (PDB codes: 1RU7 and 2IBX). After 100 models of the HA trimer were generated using MODELLER 9v6 [33], a model was chosen by a combination of the MODELLER objective function value and the discrete optimized protein energy (DOPE) statistical potential score [34]. The HA model was evaluated using PROCHECK [35] and VERIFY3D [36]. The structure of Stachyflin (CID: 493326) was downloaded from the PubChem database.

### Molecular docking

Molecular docking simulations of the HA and Stachyflin were performed using the Molegro Virtual Docker (MVD) with the default parameter settings [37].

## Competing interests

The authors declare that they have no competing interests.

## Authors’ contributions

YM drafted the manuscript and carried out *in vitro* and *in vitro* experiments in this study. MI performed the computer analyses in this study. NY and MO generated rgWSN and their mutants. TN and RY prepared the compound. MO, YS, KI, and HK participated in the coordination of the study. All authors read and approved the final manuscript.
